# Behavioral, sociodemographic, and clinical factors associated with hospital costs following road traffic injuries in Colombia

**DOI:** 10.3389/fpubh.2026.1897175

**Published:** 2026-08-25

**Authors:** Sandra Milena Zuleta Alarcón, Anderson Bermon Angarita, Alejandra Mendoza-Monsalve

**Affiliations:** 1 Department of Neurology, Physiatry Service, Hospital Internacional de Colombia, Piedecuesta, Santander, Colombia; 2 Hospital Internacional de Colombia HIC, Fundación Cardiovascular de Colombia FCV, Fundación Universitaria FCV, Piedecuesta, Santander, Colombia

**Keywords:** accidents, traffic, health care costs, multiple trauma, public health, social determinants of health

## Abstract

**Background:**

Road traffic injuries are a major public health and economic burden, particularly in low- and middle-income countries, where increasing motorization and limited trauma system capacity contribute to healthcare expenditures. Evidence on factors associated with hospital costs in Latin America remains limited.

**Objective:**

To evaluate sociodemographic, accident-related, and clinical complexity factors associated with hospital costs among patients with traffic-related injuries treated at a Colombian referral hospital.

**Methods:**

A retrospective cohort study included patients treated for road traffic injuries between 2016 and 2025 at a high-complexity referral center in Colombia. Hospital costs were expressed in Colombian pesos (COP). Multivariable linear regression using logarithmically transformed costs was performed.

**Results:**

A total of 13,899 patients were included. Median age was 32 years (IQR 25–43), and 64.0% were male. Motorcycles were involved in 87.1% of accidents. Median hospital cost was COP 431,300 (IQR 190,071–1,524,400). Surgical intervention was associated with the greatest increase in costs (1,502.6%), followed by ICU admission (1,063.2%) and each additional hospital day (6.8%). Early-morning accidents were associated with 46.8% higher costs, while nighttime crashes showed a 19.3% increase. Male sex and selected occupational categories were also associated with higher costs. After adjustment for clinical complexity markers, most geographic differences were attenuated.

**Conclusion:**

Hospital costs following road traffic injuries were mainly associated with clinical complexity markers, particularly surgery, ICU admission, and hospital length of stay. Early-morning crashes and selected sociodemographic factors were also associated with increased costs.

## Introduction

Road traffic injuries represent a substantial and growing economic challenge for healthcare systems worldwide, particularly in low- and middle-income countries (LMICs), where rapid urbanization, increasing motorization, and fragmented trauma systems amplify both mortality and healthcare expenditures (1). Beyond their clinical consequences, traffic-related injuries generate significant direct and indirect costs associated with emergency care, surgery, hospitalization, intensive care, rehabilitation, and long-term disability. Recent evidence suggests that strengthening organized trauma systems and improving access to specialized trauma care may substantially reduce preventable mortality and optimize healthcare resource utilization in LMICs (2).

Recent studies have demonstrated that hospital costs following road traffic injuries are strongly associated with behavioral, clinical, and sociodemographic determinants. Factors such as alcohol consumption, substance abuse, motorcycle-related trauma, nighttime crashes, and multisystem injuries have been associated with substantially higher healthcare expenditures and increased consumption of hospital resources (3). In particular, motorcycle users constitute one of the most vulnerable populations in LMICs due to the greater frequency of high-energy trauma, traumatic brain injury, and prolonged hospitalization (4).

In addition to injury severity, structural inequities in access to trauma care also influence the economic burden associated with road traffic injuries. Patients from geographically distant regions and underserved populations frequently experience delayed access to specialized care, higher out-of-pocket expenditures, and worse clinical outcomes (5). Recent evidence from LMICs has highlighted that deficiencies in trauma system organization, prehospital care, referral pathways, and emergency response capacity contribute not only to increased mortality but also to higher healthcare costs and inefficient resource allocation (6).

In Colombia, the sustained increase in road traffic injuries, particularly those involving motorcycles, has generated considerable pressure on emergency departments and trauma services. Although previous national studies have mainly focused on epidemiological characterization, mortality, and disability associated with traffic-related trauma, evidence regarding factors associated with hospital costs remains limited (7). A regional Colombian study identified associations between healthcare expenditures and factors such as vehicle type, injury severity, and alcohol consumption; however, evidence on the broader sociodemographic and territorial determinants of healthcare costs remains limited, particularly in high-complexity referral centers (8).

Within this context, the Hospital Internacional de Colombia (HIC), a high-complexity referral center in northeastern Colombia, receives a large volume of polytraumatized patients following road traffic injuries, particularly motorcycle-related trauma, from both urban and rural regions. Given the increasing demand for emergency and trauma care services in this setting, the institution represents an appropriate scenario to evaluate the association between accident characteristics, sociodemographic factors, and hospital healthcare costs among patients with traffic-related injuries in a middle-income country.

## Methods

### Study design and setting

A retrospective observational cohort study was conducted including patients treated for road traffic injuries at a high-complexity referral hospital in Colombia between January 2016 and December 2025. The study was reported in accordance with the Strengthening the Reporting of Observational Studies in Epidemiology (STROBE) guidelines for observational studies. The institution receives a high volume of polytraumatized patients from urban and rural areas, as well as from neighboring departments. Clinical, administrative, and cost-related information was obtained from institutional electronic medical records and hospital administrative databases.

The study was conducted at the Hospital Internacional de Colombia (HIC), located in Bucaramanga, Santander, which functions as a regional referral center for trauma and emergency care in an area characterized by high intermunicipal traffic flow.

### Eligibility criteria and patient selection

All consecutive patients treated during the study period with a confirmed diagnosis related to road traffic injuries were included. Cases were identified through institutional diagnostic coding systems and emergency department admission records. Patients of all ages and both sexes were eligible for inclusion.

Patients with incomplete clinical records for variables required in the analysis were excluded, as well as duplicate records corresponding to multiple admissions related to the same traumatic event. In cases with repeated admissions derived from the same accident, only the index hospitalization was considered to avoid correlated observations. Missing data were handled using complete-case analysis, and no imputation procedures were performed due to the retrospective nature of the study. Of the 15,126 road traffic accident records identified between 2016 and 2025, 921 were excluded due to incomplete sociodemographic information, leaving 14,205 records with complete sociodemographic characteristics. Subsequently, 226 records were excluded due to missing clinical variables, resulting in 13,979 records with complete clinical information. An additional 72 records were excluded due to incomplete cost information, and 8 records were excluded because they corresponded to repeated admissions from the same patient. Therefore, the final analytical sample included 13,899 patients, considering only the first admission.

### Variables and measurements

#### Sociodemographic variables

Sociodemographic variables included age, sex, occupation, area of origin (urban or rural), and department of origin.

#### Variables related to the road traffic injury

Accident-related variables included vehicle category, year, month, day, and time of the accident (morning, afternoon, night, and early morning). Accident years were categorized as 2016–2020, 2021, 2022, 2023, and 2024–2025 based on the distribution of eligible records across the study period. The years 2016–2020 were combined because each contributed a comparatively small number of observations, and their separate inclusion would have resulted in sparse categories and unstable estimates in the multivariable model. Although 2020 coincided with the COVID-19 pandemic and associated mobility restrictions, it was not analyzed separately because the number of eligible cases recorded during that year was insufficient to support a reliable independent estimate. The years 2021, 2022, and 2023 were retained as separate categories because they showed sufficient numbers of observations and represented the progressive recovery of road traffic and institutional referral activity. The years 2024 and 2025 were combined because they represented the most recent period and showed comparable institutional case volumes. These categories were therefore intended to improve model stability and temporal interpretability rather than to define strict pre-pandemic, pandemic, and post-pandemic periods. Hospital discharge status (alive or deceased) was also recorded.

#### Outcome variable

The primary outcome was total hospital healthcare cost, expressed in Colombian pesos (COP) adjusted to 2025 values. Cost standardization was performed using the national Consumer Price Index (CPI) for Colombia, published by the National Administrative Department of Statistics (DANE). Costs from each year were inflated to 2025 Colombian pesos using the ratio between the annual CPI for 2025 and the annual CPI corresponding to the year in which the healthcare cost was incurred. The adjustment was performed according to the following formula: adjusted cost = nominal cost × (CPI 2025 / CPI year of service). This approach allowed all costs observed during the study period to be expressed in constant 2025 COP, improving comparability across years. Operationally, healthcare costs were defined as the total direct institutional expenditures generated during hospital care, including emergency department management, diagnostic procedures, laboratory testing, imaging studies, medications, surgical interventions, hospitalization, and intensive care services when applicable. These values corresponded to direct institutional hospital costs obtained from administrative and cost-related hospital databases, rather than patient out-of-pocket payments, insurance claims, reimbursements, societal costs, or indirect costs.

Data were additionally obtained from databases stored in REDCap (9, 10).

### Statistical analysis

A descriptive univariate analysis was performed. Categorical variables were summarized using absolute frequencies and percentages, whereas continuous variables were described using means and standard deviations or medians and interquartile ranges (IQR), according to data distribution.

Normality of continuous variables was assessed using graphical methods and distribution tests. Because the primary outcome variable (healthcare cost) showed a markedly right-skewed distribution with extreme high-cost values, nonparametric methods were used for bivariate analyses. Differences in median costs between categories of independent variables were evaluated using the Mann–Whitney U test for dichotomous variables and the Kruskal–Wallis test for variables with more than two categories. Correlation between age and healthcare costs was assessed using Spearman’s correlation coefficient.

Subsequently, a multivariable linear regression model was performed using the natural logarithm of healthcare costs as the dependent variable. A linear regression model with logarithmic transformation of the dependent variable was selected because healthcare costs showed a markedly right-skewed distribution and the presence of extreme values. This transformation reduced distributional asymmetry, improved model stability, and facilitated the interpretation of regression coefficients as relative percentage changes in healthcare costs. Variables included in the multivariable model were selected according to clinical relevance and/or statistical significance in bivariate analyses. Regression coefficients (β) were transformed and expressed as percentage change in healthcare costs using the formula (e*β* − 1) × 100. Collinearity between independent variables was assessed using the Variance Inflation Factor (VIF). Model performance and goodness of fit were assessed using the coefficient of determination (R^2^), adjusted R^2^, and root mean squared error (RMSE). Effect measures were reported with corresponding 95% confidence intervals (95% CI). A two-sided *p* value <0.05 was considered statistically significant. All statistical analyses were performed using Stata software (StataCorp, College Station, TX, USA).

Given the retrospective single-center design, the study may be subject to selection and information bias related to variability in medical record completeness and institutional coding practices. However, the use of standardized institutional databases and consecutive patient inclusion was intended to minimize potential selection bias.

## Results

**Figure 1:**
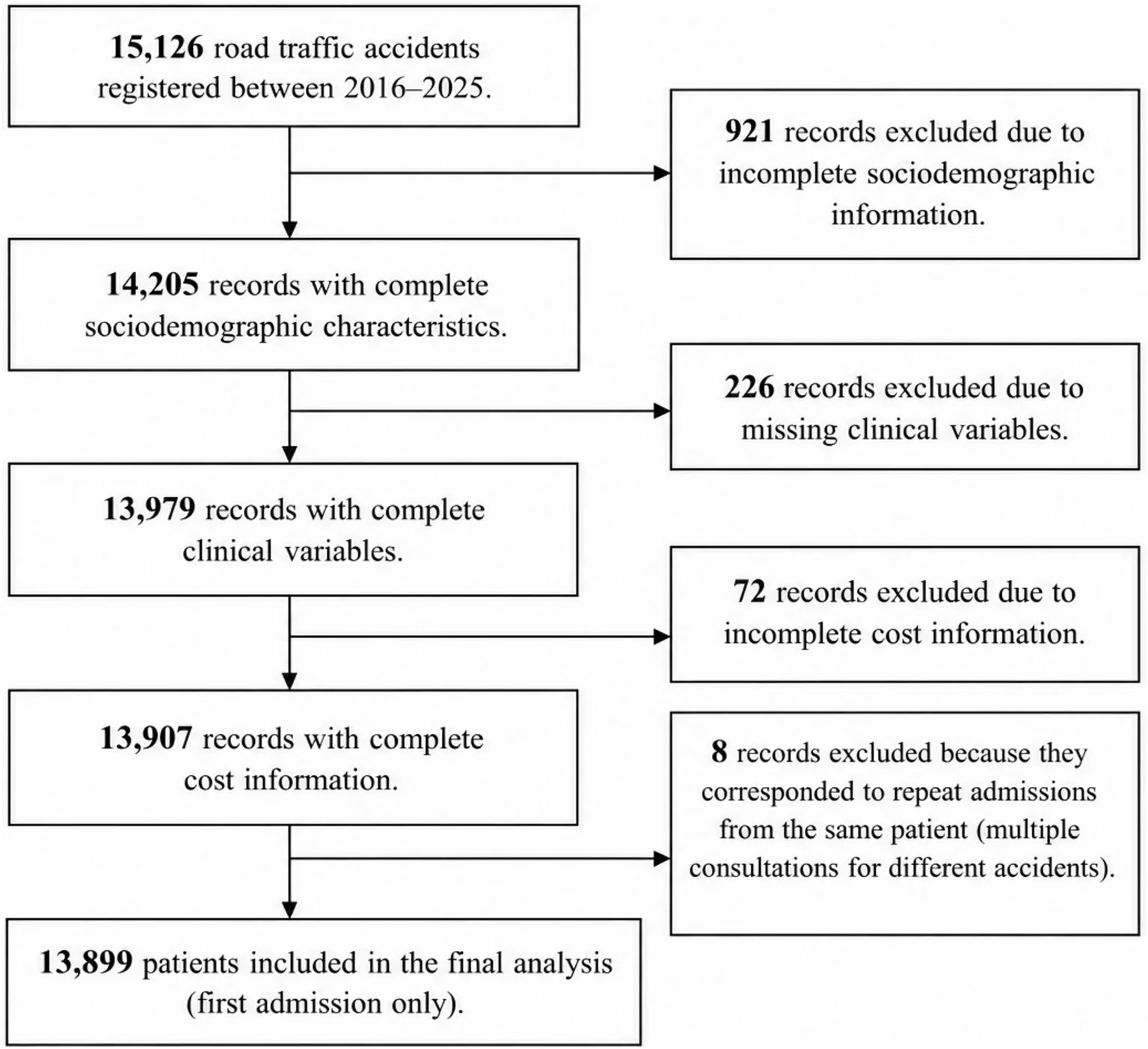
Flow diagram of patient selection for the study cohort.

A total of 13,899 patients treated for a first-time road traffic accident at a referral hospital in Colombia between 2016 and 2025 were included in the final analysis. The study cohort was derived from 15,126 road traffic injury records identified during the study period, after excluding 921 records with incomplete sociodemographic information, 226 records with missing clinical variables, 72 records with incomplete cost data, and 8 repeated admissions from the same patient. The patient selection process is presented in Figure 1.

A total of 13,899 patients treated for first-time road traffic accidents at a referral hospital in Colombia between 2016 and 2025 were included in the analysis. The median age was 32 years (IQR: 25–43), and most patients were male (64.01%). The majority of cases originated from urban areas (88.39%) and primarily involved individuals classified as homemakers, unemployed, or students (25.62%), followed by workers in commerce or self-employed occupations (24.22%). Most accidents occurred in the department of Santander (88.98%). Motorcycles were the most frequently involved vehicle category, accounting for 87.13% of cases, followed by private vehicles (10.18%). Regarding temporal distribution, the highest proportion of accidents was recorded during 2024–2025 (41.52%), with August (9.53%), September (9.36%), and October (9.32%) showing the highest frequencies. Accidents occurred more frequently during weekends, particularly on Saturdays (17.14%) and Sundays (16.91%), and predominantly during the morning (33.43%) and afternoon (31.05%). ICU admission was required in 260 patients (1.87%), and 578 patients (4.16%) underwent surgical intervention. The median hospital length of stay was 1 day (IQR: 1–1). Overall mortality was low, with 47 deaths (0.34%), while the median cost of care was COP 431,300 (IQR: 190,071–1,524,400) (Table 1).

**Table 1:** Sociodemographic and road traffic accidents characteristics.

Variable	Category	Total *n* (%)
Sociodemographic characteristics		
Age (years)	Median (IQR)	32 (25–43)
Sex	Male	8,897 (64.01)
	Female	5,002 (35.99)
Area of Origin	Urban	12,286 (88.39)
	Rural	1,613 (11.61)
Occupation	Homemaker/Unemployed/ Student	3,561 (25.62)
	Commerce/Self-employed	3,367 (24.22)
	Others	2,528 (18.19)
	Administrative/Services	2,289 (16.47)
	Professional/Technical	1,347 (9.69)
	Operational/Manual Labor	625 (4.50)
	Public Force	182 (1.31)
Related to the road traffic accident		
Department	Santander	12,367 (88.98)
	Cesar	98 (0.71)
	Norte de Santander	23 (0.17)
	Antioquia	13 (0.09)
	Other/Unclassified	1,398 (10.06)
Vehicle category	Motorcycle	12,110 (87.13)
	Private vehicle	1,415 (10.18)
	Public transportation	229 (1.65)
	Heavy cargo vehicle	130 (0.94)
	Bicycle	5 (0.04)
	Others	10 (0.07)
Year of the accident	2024–2025	5,771 (41.52)
	2023	3,029 (21.79)
	2022	2,439 (17.55)
	2021	2,490 (17.91)
	2016–2020	170 (1.22)
Month of the accident	August	1,325 (9.53)
	September	1,301 (9.36)
	October	1,296 (9.32)
	December	1,248 (8.98)
	July	1,228 (8.84)
	November	1,213 (8.73)
	May	1,188 (8.55)
	March	1,117 (8.04)
	April	1,109 (7.98)
	June	1,016 (7.31)
	February	952 (6.85)
	January	906 (6.52)
Day of the accident	Saturday	2,382 (17.14)
	Sunday	2,351 (16.91)
	Monday	1885 (13.56)
	Friday	1868 (13.44)
	Tuesday	1862 (13.40)
	Thursday	1791 (12.89)
	Wednesday	1760 (12.66)
Time of day of the accident	Morning	4,647 (33.43)
	Afternoon	4,315 (31.05)
	Night	3,724 (26.79)
	Early morning	1,213 (8.73)
Intensive care unit (ICU) admission	Yes	260 (1.87)
Surgical intervention	Yes	578 (4.16)
Hospital discharge status	Alive	13,852 (99.66)
	Deceased	47 (0.34)
Hospital length of stay (days)	Median (IQR)	1 (1–1)
Cost of care (COP)	Median (IQR)	431,300 (190,071–1′524,400)

IQR = interquartile range; COP = Colombian pesos.

**Figure 2:**
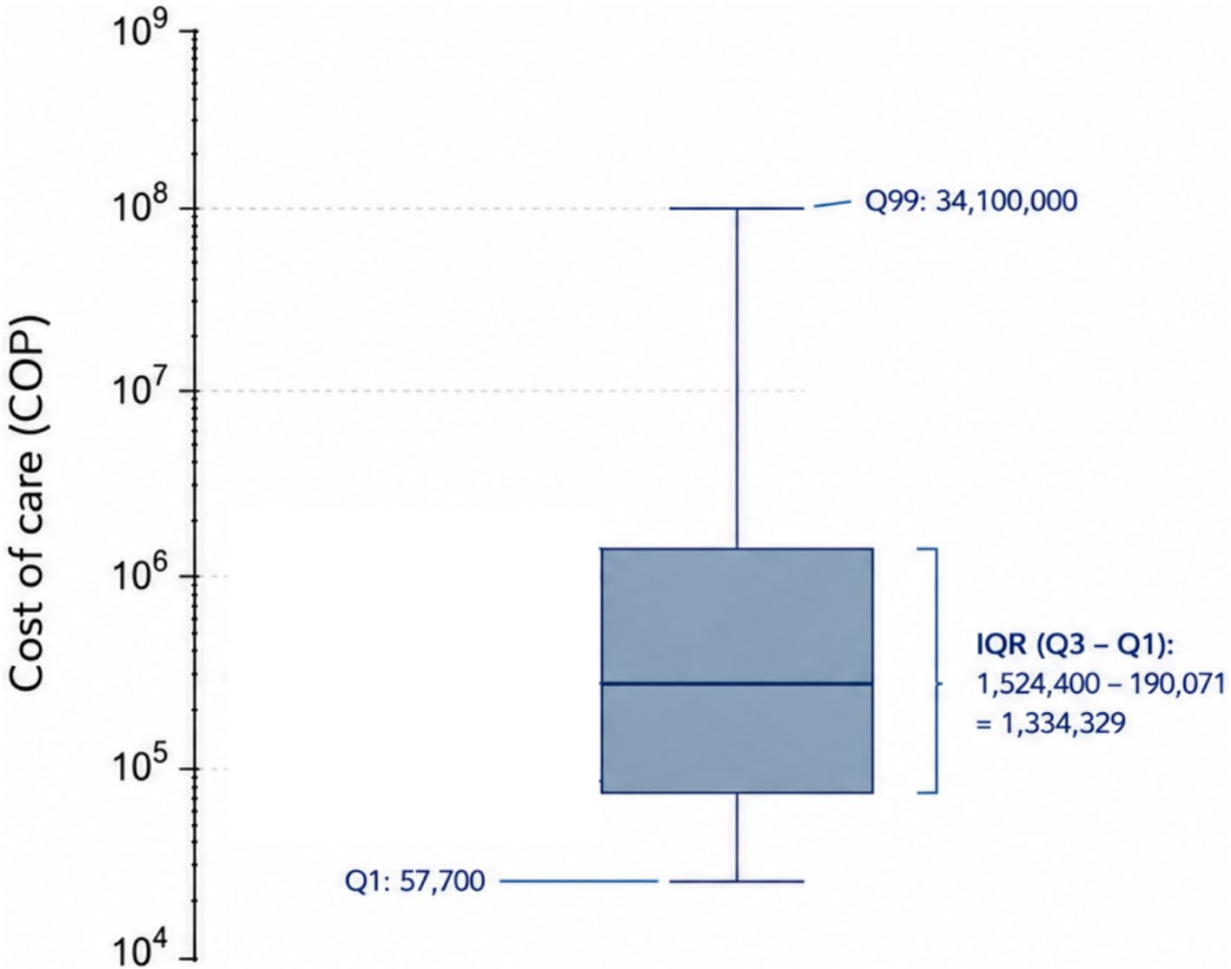
Distribution of cost care (COP). IQR = interquartile range; COP = Colombian pesos.

The overall median cost of care was COP 431,300 (IQR: 190,071–1,524,400), demonstrating substantial variability in healthcare expenditures among included patients. In addition, the cost distribution was markedly right-skewed, with extreme values approaching COP 34,100,000 at the 99th percentile, suggesting the presence of a subgroup of patients requiring significantly more complex and resource-intensive care (Figure 2).

Regarding other characteristics, a statistically significant association was observed between age and healthcare costs (Spearman’s rho = 0.026; *p* = 0.002). Concerning sociodemographic variables, men had higher median healthcare costs than women (COP 465,180 vs. COP 388,141; *p* < 0.001). Significant differences were also identified according to occupation (*p* < 0.001), with operational/manual workers presenting the highest median healthcare costs (COP 600,554; IQR: 234,102–3,002,800).

With respect to variables related to the traffic accident, significant differences in costs were identified according to the department of origin (*p* < 0.001). Patients from Norte de Santander showed the highest median costs (COP 3,013,321; IQR: 762,000–6,695,906), followed by those from Cesar (COP 992,671; IQR: 152,670–6,941,298). Significant differences were also observed according to the category of vehicle involved (*p* < 0.001). Accidents involving heavy cargo vehicles were associated with the highest median healthcare costs (COP 859,539; IQR: 235,000–4,128,730). Costs also varied significantly according to the year of the accident (*p* < 0.001), with a progressive increase over recent years and the highest costs observed during the 2024–2025 period (COP 601,641; IQR: 242,750–1,973,712).

Similarly, significant differences were found according to the month of the accident (*p* < 0.001), with December showing the highest median healthcare costs (COP 681,700; IQR: 235,148–2,642,498). According to the day of occurrence, the highest costs were observed on Sundays (COP 494,425; IQR: 205,671–2,035,216) and Saturdays (COP 471,328; IQR: 189,200–1,905,837) (*p* < 0.001). Regarding the time of occurrence, accidents taking place during the early morning hours were associated with the highest median costs (COP 730,886; IQR: 232,000–2,887,195), with statistically significant differences across time periods (*p* < 0.001).

Finally, patients admitted to the ICU had markedly higher median healthcare costs than those not requiring ICU care (COP 11,212,150; IQR: 7,211,470–21,452,669; *p* < 0.001). Similarly, patients who underwent surgical intervention showed substantially higher median costs (COP 11,055,610; IQR: 6,740,935–19,543,498; *p* < 0.001). Hospital length of stay was also positively correlated with healthcare costs (Spearman’s rho = 0.518; *p* < 0.001). Likewise, patients who died during hospitalization incurred significantly higher costs compared with survivors (COP 5,223,285 vs. COP 429,458; *p* < 0.001) (Table 2).

**Table 2:** Costs according to sociodemographic characteristics and variables related to road traffic accidents.

Variable	Category	Cost median (IQR)	*p*-Value
Sociodemographic characteristics			
Age (years)	Median	431.300 (190.071–1.524.400)	0.002
Sex	Male	465.180 (199.254–1.839.601)	<0.001
	Female	388.141 (179.761–1.162.300)	
Area of Origin	Urban	433.648 (189.944–1.542.268)	0.437
	Rural	417.510 (190.200–1.406.941)	
Occupation	Homemaker/Unemployed/ Student	498.195 (201.157–1.821.720)	<0.001
	Commerce/Self-employed	486.154 (206.756–1.857.190)	
	Others	393.903 (169.050–1.262.546)	
	Administrative/Services	366.462 (183.308–1.060.584)	
	Professional/Technical	370.000 (179.032–978.600)	
	Operational/Manual Labor	600.554 (234.102–3.002.800)	
	Public Force	467.689 (216.773–1.563.299)	
Related to the road traffic accident			
Department of Origin	Santander	417.440 (189.116–1.372.302)	<0.001
	Cesar	992.671 (152.670–6.941.298)	
	Norte de Santander	3.013.321 (762.000–6.695.906)	
	Antioquia	810.585 (542.392–976.461)	
	Other/Unclassified	650.528 (196.400–3.318.838)	
Vehicle Category	Motorcycle	430.849 (194.531–1.487.100)	<0.001
	Private vehicle	456.200 (162.064–1.727.342)	
	Public transportation	297.610 (148.906–1.122.200)	
	Heavy cargo vehicle	859.539 (235.000–4.128.730)	
	Bicycle	365.340 (252.860–449.431)	
	Others	325.745 (210.955–461.592)	
Year of the accident	2024–2025	601.641 (242.750–1.973.712)	<0.001
	2023	577.250 (274.275–2.173.920)	
	2022	309.538 (166.870–1.057.215)	
	2021	246.862 (131.731–646.977)	
	2016–2020	52.400 (52.400–52.400)	
Month of the accident	August	371.309 (185.036–1.148.344)	<0.001
	September	447.049 (197.571–1.720.006)	
	October	568.800 (219.304–1.741.585)	
	December	681.700 (235.148–2.642.498)	
	July	437.756 (191.707–1.468.487)	
	November	478.024 (186.590–1.880.095)	
	May	376.950 (182.600–1.214.913)	
	March	372.317 (183.490–1.304.880)	
	April	372.593 (186.300–1.263.400)	
	June	426.417 (187.508–1.295.622)	
	February	366.216 (169.950–1.121.307)	
	January	394.150 (179.869–1.300.949)		
Day of the accident	Saturday	471.328 (189.200–1.905.837)	<0.001
	Sunday	494.425 (205.671–2.035.216)	
	Monday	422.992 (184.878–1.608.200)	
	Friday	426.281 (188.712–1.254.465)	
	Tuesday	439.943 (197.465–1.354.300)	
	Thursday	412.000 (188.830–1.322.033)	
	Wednesday	366.045 (179.802–1.149.972)	
Time of day of the accident	Morning	370.000 (179.024–1.128.088)	<0.001
	Afternoon	427.868 (188.900–1.505.065)	
	Night	485.721 (202.766–1.682.467)	
	Early morning	730.886 (232.000–2.887.195)	
Intensive Care Unit (ICU) admission	Yes	11.212.150 (7.211.470–21.452.669)	<0.001
Surgical intervention	Yes	11.055.610 (6.740.935–19.543.498)	<0.001
Hospital length of stay (days)	Median (IQR)	431.300 (190.071–1.524.400)	<0.001
Hospital discharge status	Alive	429.458 (189.769–1.509.990)	<0.001
	Deceased	5.223.285 (1.162.060–11.400.000)	

IQR = interquartile range; CI = confidence interval.

In the multivariable linear regression model using the logarithm of healthcare costs as the dependent variable, available markers of clinical complexity showed the strongest associations with healthcare costs. Surgical intervention was associated with the largest increase in healthcare costs, corresponding to an approximately 1,502.6% increase compared with patients who did not undergo surgery (*β* = 2.774; 95% CI: 2.661–2.887; *p* < 0.001). Similarly, ICU admission was associated with an approximately 1,063.2% increase in healthcare costs (*β* = 2.454; 95% CI: 2.284–2.623; *p* < 0.001). Each additional day of hospitalization was associated with a 6.8% increase in healthcare costs (*β* = 0.066; 95% CI: 0.061–0.071; *p* < 0.001).

Concerning the timing of the accident, events occurring during early morning hours remained associated with higher healthcare costs, with a 46.8% increase compared with morning accidents (*β* = 0.384; 95% CI: 0.299–0.469; *p* < 0.001). Accidents occurring during nighttime and afternoon hours were associated with cost increases of 19.3% (*β* = 0.176; 95% CI: 0.118–0.234; *p* < 0.001) and 8.1% (*β* = 0.078; 95% CI: 0.022–0.134; *p* = 0.006), respectively.

Regarding sociodemographic characteristics, male sex was associated with a 15.0% increase in healthcare costs (*β* = 0.140; 95% CI: 0.091–0.189; *p* < 0.001). Operational/manual occupation was associated with 34.7% higher costs (*β* = 0.298; 95% CI: 0.167–0.428; *p* < 0.001), while patients categorized as homemakers, unemployed, or students showed a 26.1% increase (*β* = 0.232; 95% CI: 0.147–0.317; *p* < 0.001), and those in commerce or self-employed occupations showed a 19.4% increase (*β* = 0.177; 95% CI: 0.091–0.264; *p* < 0.001). Each additional year of age was associated with a 0.6% increase in healthcare costs (*β* = 0.006; 95% CI: 0.004–0.008; *p* < 0.001).

After adjustment for clinical complexity markers, most geographic differences were attenuated. Patients from other or unclassified departments had 18.0% higher healthcare costs than those from Santander (*β* = 0.166; 95% CI: 0.107–0.225; *p* < 0.001), whereas the associations for Cesar, Norte de Santander, and Antioquia were no longer statistically significant. Regarding vehicle category, public transportation was associated with 28.2% lower healthcare costs (*β* = −0.332; 95% CI: −0.509 to −0.154; *p* < 0.001), while private vehicles and heavy cargo vehicles were not statistically significant in the adjusted model. The final multivariable model was statistically significant overall (*F* = 218.66; *p* < 0.001) and showed an R^2^ of 0.2744, an adjusted R^2^ of 0.2732, and an RMSE of 1.3385. No relevant multicollinearity was observed after including ICU admission, surgical intervention, and hospital length of stay, with a mean VIF of 1.34 (Table 3).

**Table 3:** Multivariable linear regression analysis of factors associated with healthcare costs adjusted for clinical complexity markers.

Variable	β coefficient (95% CI)	Change in cost (%)	p-value
Age (years)	0.006 (0.004–0.008)	0.6	<0.001
Sex (Ref. Female)	0.140 (0.091–0.189)	15.0	<0.001
Occupation (Ref. Professional/Technical)			
Administrative/Services	−0.001 (−0.093–0.090)	−0.1	0.975
Operational/Manual labor	0.298 (0.167–0.428)	34.7	<0.001
Commerce/Self-employed	0.177 (0.091–0.264)	19.4	<0.001
Homemaker/Unemployed/Student	0.232 (0.147–0.317)	26.1	<0.001
Public Force	0.155 (−0.054–0.363)	16.7	0.147
Others	−0.007 (−0.097–0.084)	−0.7	0.882
Department of origin (Ref. Santander)			
Cesar	0.085 (−0.209–0.380)	8.9	0.571
Norte de Santander	0.357 (−0.160–0.874)	43.0	0.175
Antioquia	0.730 (−0.343–1.802)	107.4	0.182
Other/Unclassified	0.166 (0.107–0.225)	18.0	<0.001
Vehicle category (Ref. Motorcycle)			
Private vehicle	−0.072 (−0.147–0.003)	−7.0	0.060
Public transportation	−0.332 (−0.509–-0.154)	−28.2	<0.001
Heavy cargo vehicle	0.192 (−0.042–0.426)	21.1	0.108
Bicycle	−0.121 (−1.295–1.053)	−11.4	0.839
Others	0.030 (−0.801–0.861)	3.0	0.944
Time of day of accident (Ref. Morning)			
Afternoon	0.078 (0.022–0.134)	8.1	0.006
Night	0.176 (0.118–0.234)	19.3	<0.001
Early morning	0.384 (0.299–0.469)	46.8	<0.001
ICU admission (Ref. No ICU admission)	2.454 (2.284–2.623)	1063.2	<0.001
Surgical intervention (Ref. No surgical intervention)	2.774 (2.661–2.887)	1502.6	<0.001
Hospital length of stay, per additional day	0.066 (0.061–0.071)	6.8	<0.001

CI = confidence interval; ICU = intensive care unit.

## Discussion

In this large retrospective cohort of 13,899 patients treated for road traffic injuries at a Colombian referral hospital, healthcare costs were associated with clinical complexity, sociodemographic characteristics, occupational profile, accident timing, and selected territorial factors. After adjusting for available markers of injury severity and hospital resource utilization, surgical intervention, ICU admission, and hospital length of stay emerged as the strongest factors associated with increased costs. Although some sociodemographic variables reached statistical significance, their magnitude of association was comparatively smaller and should therefore be interpreted with caution in terms of clinical and policy relevance. These findings reinforce that the economic burden of traffic-related trauma is driven not only by the occurrence of the crash itself, but also by the downstream need for high-complexity hospital care, including surgical management, critical care, and prolonged hospitalization (11). This is consistent with evidence from trauma and rehabilitation cost studies showing that patients requiring intensive multidisciplinary management generate substantially higher expenditures and longer healthcare utilization trajectories (12).

The inclusion of ICU admission, surgical intervention, and hospital length of stay strengthened the interpretation of the model by partially accounting for clinical complexity. Surgical intervention and ICU admission were associated with the largest increases in healthcare costs, while each additional hospital day was also independently associated with higher expenditure. These findings are clinically plausible, as operative care, critical care monitoring, advanced diagnostics, procedures, medications, and prolonged bed occupancy represent major components of trauma-related hospital costs. From a health systems perspective, these results suggest that strategies aimed at preventing severe injuries and improving early triage, timely referral, and efficient in-hospital care pathways may have important implications for reducing the economic burden of road traffic injuries (3).

The associations identified for male sex, operational/manual occupations, commerce or self-employment, homemaker/unemployed/student status, and nighttime or early-morning crashes should be interpreted within a broader social determinants framework. These factors may reflect differential exposure to road traffic, transportation dependence, informal labor conditions, occupational vulnerability, and gender-associated risk behaviors (13). Evidence from Colombian professional drivers has shown that job strain and driving styles may be associated with involvement in work-related traffic crashes, supporting the potential relevance of occupational conditions and behavioral patterns in road safety (14). However, these mechanisms were not directly measured in the present study and should therefore be considered plausible contextual explanations rather than established causal pathways. Compared with clinical complexity markers, the magnitude of several sociodemographic associations was more modest, indicating that these variables may be more useful for identifying vulnerable groups than for explaining the largest cost increases. Importantly, early-morning crashes remained significantly associated with higher costs even after adjustment for available clinical complexity markers, suggesting that time of occurrence may capture additional risk conditions such as fatigue, reduced visibility, speeding, limited traffic control, delayed access to care, or increased likelihood of impaired driving. Similar epidemiological patterns have been reported in LMICs, where men, motorcycle users, and nighttime crashes consistently account for a disproportionate share of severe road traffic trauma (15).

Geographic differences in healthcare costs were attenuated after adjustment for ICU admission, surgery, and hospital length of stay. Although patients from Cesar and Norte de Santander showed higher crude costs, these associations were no longer statistically significant in the fully adjusted model. This pattern suggests that part of the higher costs observed among patients from more distant regions may be related to referral dynamics and the clinical complexity of cases requiring care at a high-complexity trauma center. Longer transport times, delayed access to definitive care, and referral of patients requiring specialized surgical or critical care services may contribute to higher resource utilization. Therefore, geographic origin should be interpreted as a contextual factor that may reflect distance to specialized care, regional referral pathways, and differences in access to timely trauma services. These findings support the importance of strengthening coordinated referral networks, prehospital transport, and timely access to high-complexity trauma care for patients from distant regions (16).

Finally, several associations, although statistically significant, should be interpreted in terms of their clinical and policy relevance. For example, age showed a statistically significant but modest association with healthcare costs, corresponding to an estimated 0.6% increase in costs per additional year of age, whereas surgical intervention, ICU admission, and hospital length of stay showed substantially larger cost differences (17). This distinction is important in large datasets, where small effects may reach statistical significance without necessarily representing factors with major clinical or economic relevance. Accordingly, the interpretation of the model should prioritize effect size and practical implications rather than statistical significance alone. From a policy perspective, the most relevant findings are those related to high-complexity care, early-morning crashes, and vulnerable occupational and sociodemographic groups. These results may inform targeted prevention strategies, trauma system planning, and hospital resource allocation in middle-income settings (18).

### Limitations

This study included a large sample of patients treated over nearly a decade in a high-complexity referral center, allowing the evaluation of factors associated with healthcare costs in a real-world hospital setting. However, its retrospective single-center design may limit generalizability to other institutions or regions with different trauma care organization, referral pathways, and cost structures. As with studies based on routinely collected data, some relevant indicators of crash circumstances and injury severity, including alcohol or substance use, protective device use, distraction, speeding, and standardized trauma severity scores, were not consistently available. Although ICU admission, surgical intervention, and hospital length of stay provided useful markers of clinical complexity, they may not fully capture injury severity. Costs were limited to direct institutional hospital costs, and therefore indirect costs, out-of-pocket expenditures, prehospital costs, and broader societal costs. Future multicenter studies incorporating standardized severity measures and more comprehensive cost data are warranted.

## Conclusion

Hospital costs associated with road traffic injuries were mainly associated with markers of clinical complexity, including surgical intervention, ICU admission, and hospital length of stay. Early morning crashes, male sex, selected occupational categories, and residence in other or unclassified departments were also associated with higher healthcare costs. These findings suggest that the economic burden of road traffic trauma is closely related to the need for high-complexity hospital care and may inform targeted prevention strategies, trauma system planning, and hospital resource allocation in middle-income settings.

## Data Availability

The datasets presented in this article are not readily available because the dataset contains clinical and administrative information derived from institutional medical records and is subject to ethical, legal, and confidentiality restrictions. Due to patient privacy considerations and institutional policies, the data are not publicly available. Requests to access the datasets should be directed to mmendoza806@unab.edu.co.
